# Rice pest detection via multi-scale edge network and wavelet attention enhancement

**DOI:** 10.3389/fpls.2026.1748419

**Published:** 2026-02-17

**Authors:** Xinyue Huang, Ruoxuan Zhou

**Affiliations:** School of Software Engineering, Jiangxi University of Science and Technology, Nanchang, China

**Keywords:** deep learning, edge feature enhancement, object detection, rice pest detection, wavelet attention mechanism

## Abstract

Rice pest detection faces critical challenges including small target recognition difficulties, high morphological similarities, and complex field backgrounds. This study proposes BEAM-YOLO (Bi-branch Edge Attention Multi-scale YOLO) to address these limitations.We constructed the JRICE-PD dataset encompassing 11 economically significant rice pests (4,565 images) and developed four innovative modules: a Multi-scale morphological Edge Network (MEN) enhancing feature discrimination; a Bi-branch Attention Feature Enhancement (BAFE) module utilizing Haar wavelet transform for foreground-background separation; an Enhanced Multi-scale Bidirectional Feature Pyramid Network (EM-BFPN) optimizing information interaction; and a Spatial-Channel Augmented Upsampling (SCAU) improving small target detection.BEAM-YOLO achieves 86.6±0.5% mAP@50 and 72.7±0.9% mAP@50-95, outperforming YOLOv11 by 3.3% and 3.0% respectively, while maintaining relatively low computational overhead and parameter count. This research provides reliable algorithmic support for intelligent agricultural pest monitoring systems, contributing to precision agriculture advancement and application.

## 1 Introduction

Rice, as one of the world’s most crucial food crops, provides a fundamental source of nutrition for more than half the global population and plays a vital role in maintaining global food security (Bailey-Serres et al., 2019). However, rice production faces severe threats from pests and diseases. According to statistics from the Food and Agriculture Organization (FAO), annual global crop losses due to pests are estimated at 20-40% (Rizzo et al., 2021). Therefore, timely and accurate identification and detection of rice pests and diseases is of significant importance for ensuring food security, improving agricultural production efficiency, reducing pesticide use, decreasing environmental pollution, and promoting sustainable agricultural development (Li S. et al., 2022).

Traditional rice pest and disease detection primarily relies on the experiential judgment of agricultural experts and field surveys. This approach is limited in scope, time-consuming, labor-intensive, and inherently subjective (Jiang et al., 2008). Specifically, conventional methods include manual field inspection, yellow sticky trap attraction, light trapping techniques, and laboratory microscopic examination (Thenmozhi and Reddy, 2019). Though these traditional methods have certain value under specific conditions, they generally suffer from low efficiency, unstable precision, and difficulties in large-scale automation, making them inadequate for meeting the precise and intelligent development needs of modern agriculture (Preti et al., 2021).

In recent years, numerous classical deep learning architectures such as AlexNet, VGGNet, Inception, ResNet, and DenseNet have been applied to agricultural pest detection (Li et al., 2020; Lu et al., 2025a). These methods can automatically extract hierarchical features from images without requiring manually designed feature extraction algorithms, significantly improving detection accuracy and efficiency. For instance, Hasan et al (Hasan et al., 2019). proposed an integrated approach combining support vector machines with deep convolutional neural networks, which markedly improved the accuracy of rice pest and disease recognition.

Object detection-based methods have become increasingly widespread across various fields and, due to their advantages in real-time performance and localization accuracy, have emerged as one of the mainstream approaches for rice pest and disease detection (Venkateswara and Padmanabhan, 2025). Object detection technology not only identifies the types of pests and diseases in images but also precisely locates their positions, enabling the possibility of precision pesticide application. As a leading method in object detection research, the YOLO series has inspired numerous improvements. Zheng et al (Zheng et al., 2024). proposed Rice-YOLO, a lightweight pest detection algorithm based on YOLOv5. This model, built upon YOLOv8-N, incorporates an efficient detection head designed for the complex characteristics of pests, while introducing deep supervision techniques and an improved dynamic upsampling module, achieving excellent detection performance on the large-scale public IP102 pest dataset and R2000 dataset.Lu et al (Lu et al., 2025b). developed IMobileTransformer, a fusion-based lightweight model that integrates MobileNet’s local feature extraction with Transformer’s global dependency modeling through a three-branch architecture, demonstrating the effectiveness of hybrid lightweight architectures for rice disease identification. Xiong et al (Xiong et al., 2024). addressed the problem of rice pest detection in complex agricultural environments by optimizing the YOLOv8 model, introducing the CBAM (Convolutional Block Attention Module) attention mechanism and BiFPN (Bidirectional Feature Pyramid Network) for feature fusion, significantly improving detection precision in complex agricultural environments.

Despite significant progress in YOLO-based rice pest and disease detection, several challenges persist in practical applications. Firstly, conventional convolutional structures struggle to extract detailed features of minute pests, and increasing network depth leads to degradation of edge information, affecting the ability to distinguish morphologically similar pests. Existing models have not achieved optimal balance between computational efficiency and detection precision (Chakrabarty et al., 2024). Secondly, the low contrast between pests and complex field backgrounds causes feature confusion problems, particularly under non-uniform lighting and occlusion conditions. Single spatial attention mechanisms cannot simultaneously capture multi-scale morphological variations and low-contrast features, resulting in detection instability (Wang et al., 2025). Finally, traditional feature pyramid networks lack adaptive fusion capabilities for multi-scale features and fail to differentiate contribution degree variations among different resolution features, making individual recognition difficult in densely distributed scenarios and limiting overall detection performance (Prasath and Akila, 2023).

To address these challenges, this paper proposes an improved YOLOv11 (Khanam and Hussain, 2024) model—Bifurcated Edge-Attention Multi-scale YOLO (BEAM-YOLO)—for high-precision real-time detection of rice pests and diseases. The main contributions of this research are as follows:

1. We constructed a high-quality rice pest dataset named JRICE-PD, comprising 4,565 images across 11 economically significant pest species. The dataset integrates multi-source acquisitionwith a three-tier expert review mechanism, ensuring both ecological authenticity and annotation consistency.
2. We propose the Morphological Edge Network (MEN) module to address edge information degradation in deep networks. Through multi-scale adaptive pooling and an EdgeEnhancer mechanism, MEN effectively captures fine morphological features of minute pests and enhances discrimination capability for morphologically similar species.
3. We design the Bifurcated Attention Feature Enhancement (BAFE) module to resolve foreground-background feature confusion. By employing Haar wavelet transform for frequency domain decomposition and a cascaded dual-attention mechanism, BAFE effectively separates pest targets from complex agricultural backgrounds under varying lighting and occlusion conditions.
4. We propose an Enhanced Multi-scale Bidirectional Feature Pyramid Network (EM-BFPN) to overcome the limitations of traditional FPNs in adaptive feature fusion. The Adaptive Feature Fusion Mechanism (AFFM) dynamically adjusts feature contributions across scales, while the Multi-scale Convolution Module (MSCM) enables gradient receptive field coverage for improved detection in dense distribution scenarios.
5. We introduce a Spatial-Channel Augmented Upsampling (SCAU) module that combines channel shuffling with Multi-Directional Feature Shifting (MDFS) to enhance small target detection sensitivity without increasing computational overhead.

## 2 Materials and methods

### 2.1 Data collection

This study established a rice pest dataset named JRICE-PD. The data collection site was located in Nanchang City, Jiangxi Province (28°40′~29°05′N, 115°45′~116°15′E), one of China’s major rice-growing regions (Yang et al., 2021).

**Figure 1:**
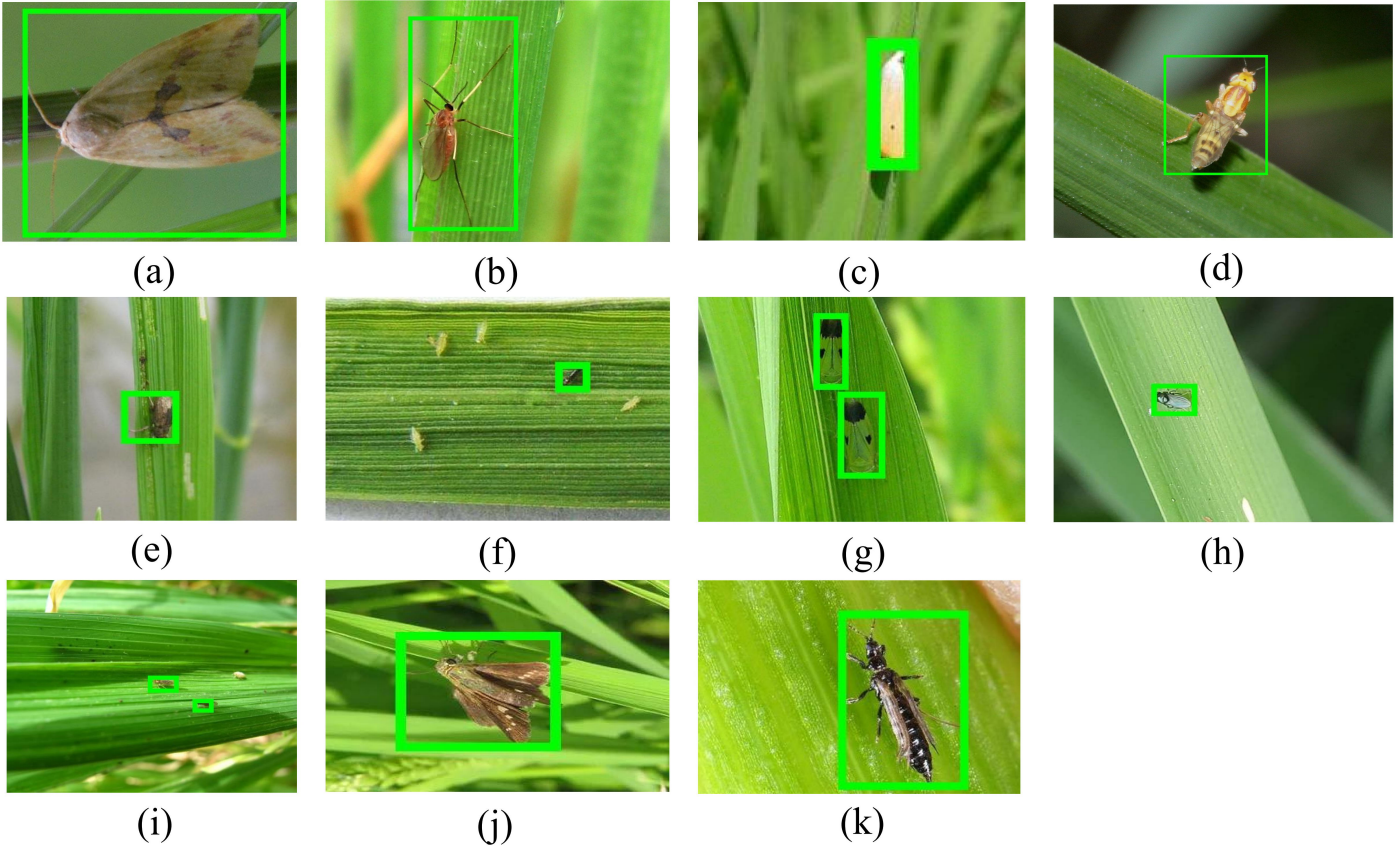
Rice pest species, where **(A)** is Delphacidae, **(B)** is Crambidae, **(C)** is Cecidomyiidae, **(D)** is Phlaeothripidae, **(E)** is Thripidae, **(F)** is Chloropidae, **(G)** is Ephydridae, **(H)** is Cicadellidae, **(I)** is Noctuidae, **(J)** is Hesperiidae, **(K)** is Curculionidae.

**Figure 2:**
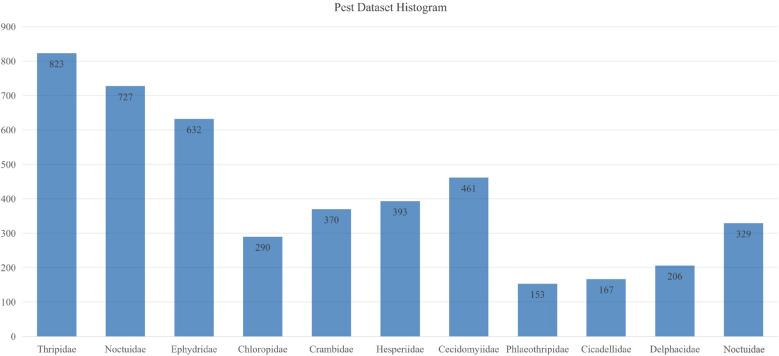
Bar chart showing specific quantities of pest species in the dataset.

The dataset encompasses 11 common and destructive rice pests, as shown in **Figure 1**: Curculionidae, Delphacidae, Cicadellidae, Phlaeothripidae, Cecidomyiidae, Hesperiidae, Crambidae, Chloropidae, Ephydridae, Noctuidae, and Thripidae. Field data collection was conducted between June and September 2024 across approximately 50 hectares of paddy fields, covering the rice growing season from tillering to maturation stages. Images were captured using iPhone 13 (12MP, f/1.6 aperture) at shooting distances of 10–50 cm, yielding 2,164 original images at 4032×3024 pixels resolution. Camera settings included auto-focus with exposure compensation of -1.0 to +1.0 EV for varying lighting conditions. Additionally, 2,401 supplementary images were collected from online resources including Google Scholar and Baidu Images, constructing a comprehensive dataset of 4,565 images (**Figure 2**). All images were resized to 640×640 pixels for training.

**Figure 3:**
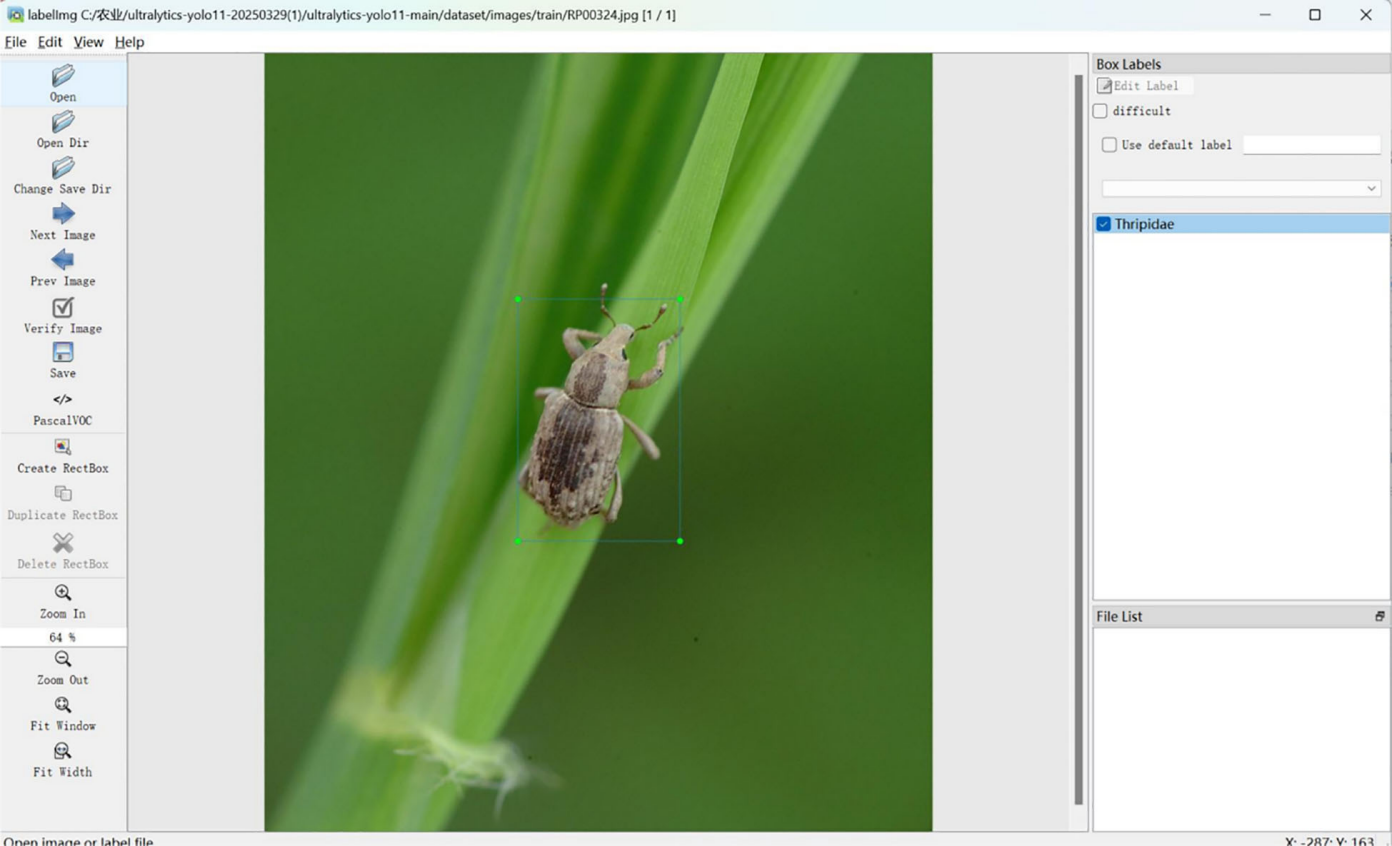
Dataset annotation process using the LabelImg tool.

Data annotation employed the LabelImg tool following YOLO format with a “tight bounding box” principle (**Figure 3**). An annotation team comprising two entomological experts and three trained annotators performed annotations under a three-tier quality control mechanism. Annotation consistency was quantified using Intersection over Union (IoU) between independent annotations on 200 randomly sampled images, achieving a mean IoU of 0.91. Annotations with IoU below 0.85 were re-annotated until consensus was reached.

The dataset was divided into training (3,652), validation (456), and test (457) sets at an 8:1:1 ratio.

### 2.2 YOLOv11 baseline framework

**Figure 4:**
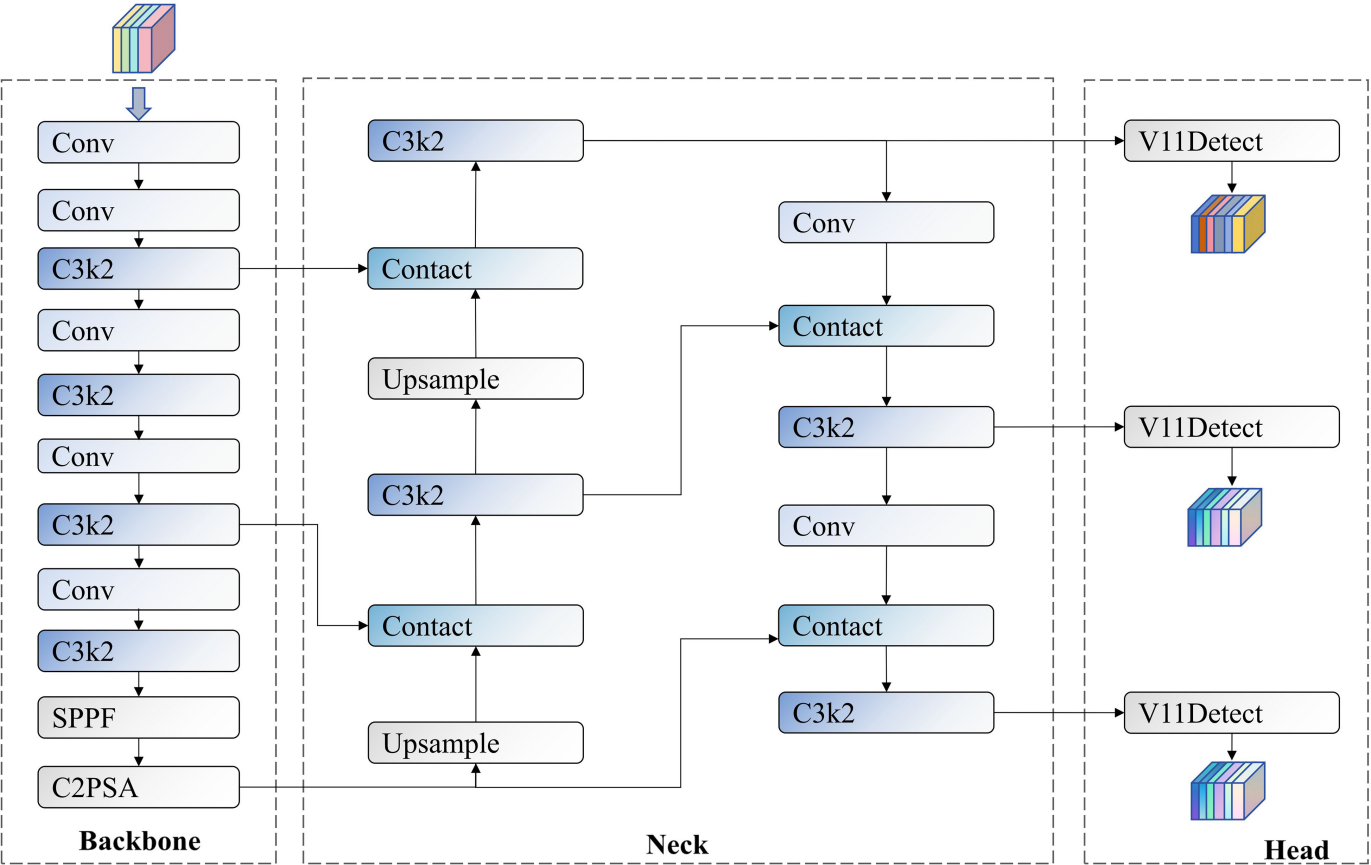
YOLOv11 network structure diagram.

YOLOv11 represents one of the most advanced single-stage object detection algorithms currently available, incorporating multiple improvements in network structure and feature extraction compared to previous versions. The basic architecture of YOLOv11 comprises three main components: Backbone, Neck, and Head, with its network structure illustrated in **Figure 4**.

The backbone network is responsible for extracting multi-scale features from input images. It first downsamples the image using initial convolutional layers, then generates feature maps of different resolutions through stacked convolutional layers and specialized modules. The neck network aggregates multi-scale feature maps from the backbone network, fusing and enhancing features before passing them to the detection head. It primarily consists of multiple convolutional layers, C3k2 blocks, Concat operations, and upsampling modules. The neck network first upsamples the low-level features (P5) processed by SPPF and C2PSA to the size of mid-level features (P4) and connects them with P4 features; it then upsamples the connected features to the size of high-level features (P3) and connects them.

The detection head is the final component of the model, responsible for generating prediction results. It receives three features from the neck network, corresponding to high-level, mid-level, and low-level features. The detection head utilizes these three features for focal loss calculation, bounding box detection, and class detection. This design enables YOLOv11 to achieve efficient and accurate object localization and classification in application scenarios.

## 3 Methods

### 3.1 BEAM-YOLO network structure

**Figure 5:**
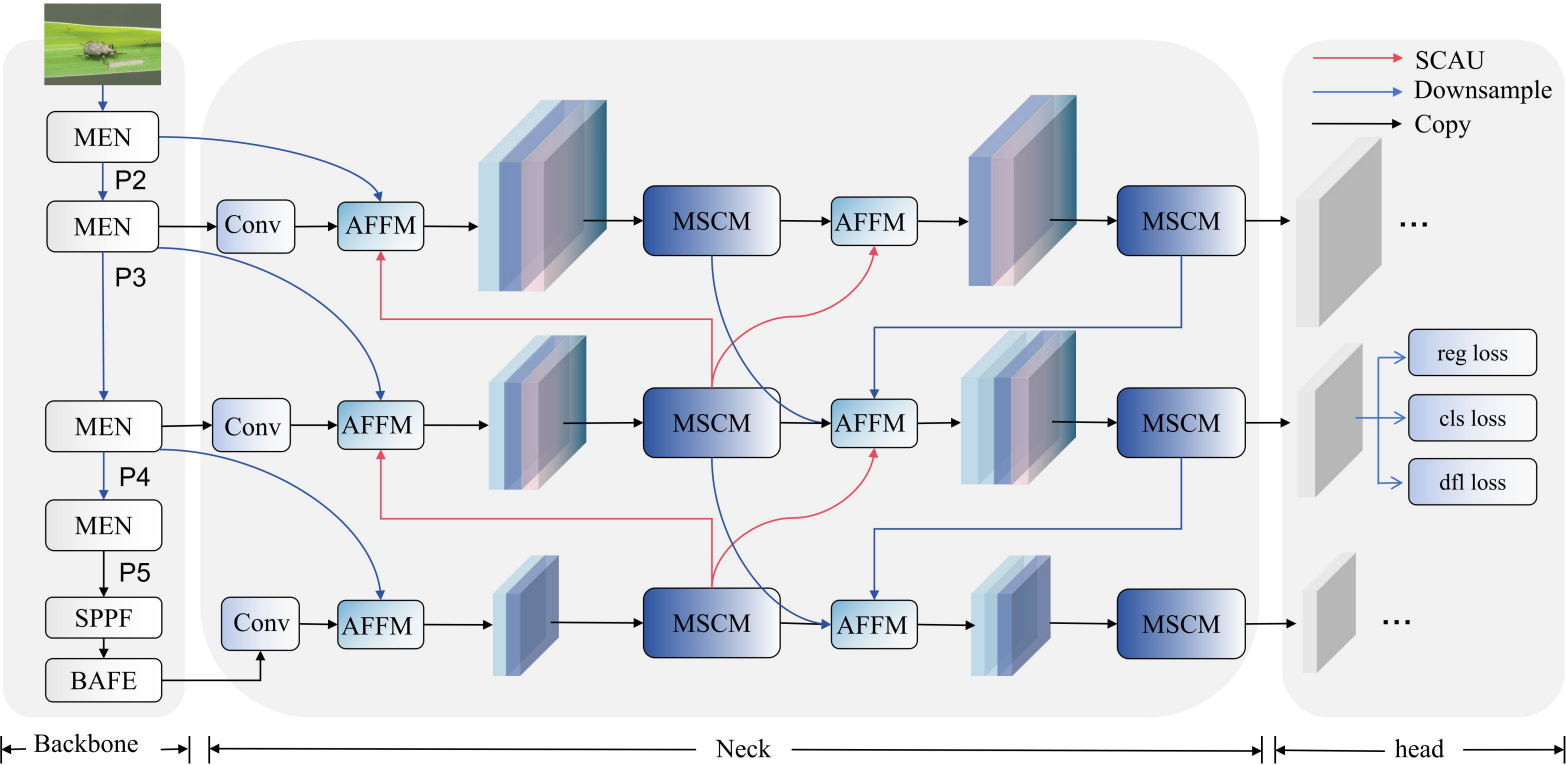
BEAM-YOLO network structure.

The BEAM-YOLO network architecture proposed in this study is algorithmically optimized for rice pest detection tasks, establishing an efficient and precise end-to-end detection algorithm through the synergistic action of four innovative modules. The workflow of BEAM-YOLO can be divided into three key stages: feature extraction, feature fusion, and multi-scale detection, with the overall network structure shown in **Figure 5**.

In the feature extraction stage, the network first establishes initial feature representation through standard convolutional layers, followed by deployment of the MEN module for feature enhancement. The MEN module constructs multi-scale feature representation through the MSF structure while simultaneously reinforcing edge feature extraction, significantly improving the discriminative ability for morphologically similar pests. The Bifurcated Attention Feature Enhancement (BAFE) module integrated at the backbone network terminus utilizes Haar wavelet transform to decompose features into foreground and background components, and establishes contrast enhancement effects through a dual attention mechanism, effectively resolving foreground-background feature confusion problems in complex field environments.

The feature fusion stage employs the EM-BFPN structure to bidirectionally fuse P3, P4, and P5 three-level features extracted by the backbone. EM-BFPN adopts top-down transmission of high-2level semantic information and bottom-up aggregation of refined spatial details. The network integrates the MSCM module for feature processing, which applies convolution kernel combinations of {1,3,5}, {3,5,7}, and {5,7,9} respectively according to the receptive field requirements of different feature levels, forming a gradient multi-scale feature expression. In the feature upsampling process, the innovative SCAU module is employed, significantly expanding the receptive field range without increasing computational burden through depth-separable convolution, channel shuffling, and multi-directional spatial shifting operations.

In the multi-scale detection stage, the network constructs a highly optimized detection head structure, outputting detection results in parallel at P3, P4, and P5 feature levels, achieving precise localization and classification of pest targets of different scales. The three feature levels work collaboratively, forming a detection range covering multiple scales, effectively addressing the detection challenges posed by significant size variations among rice pests.

### 3.2 MEN module

**Figure 6:**
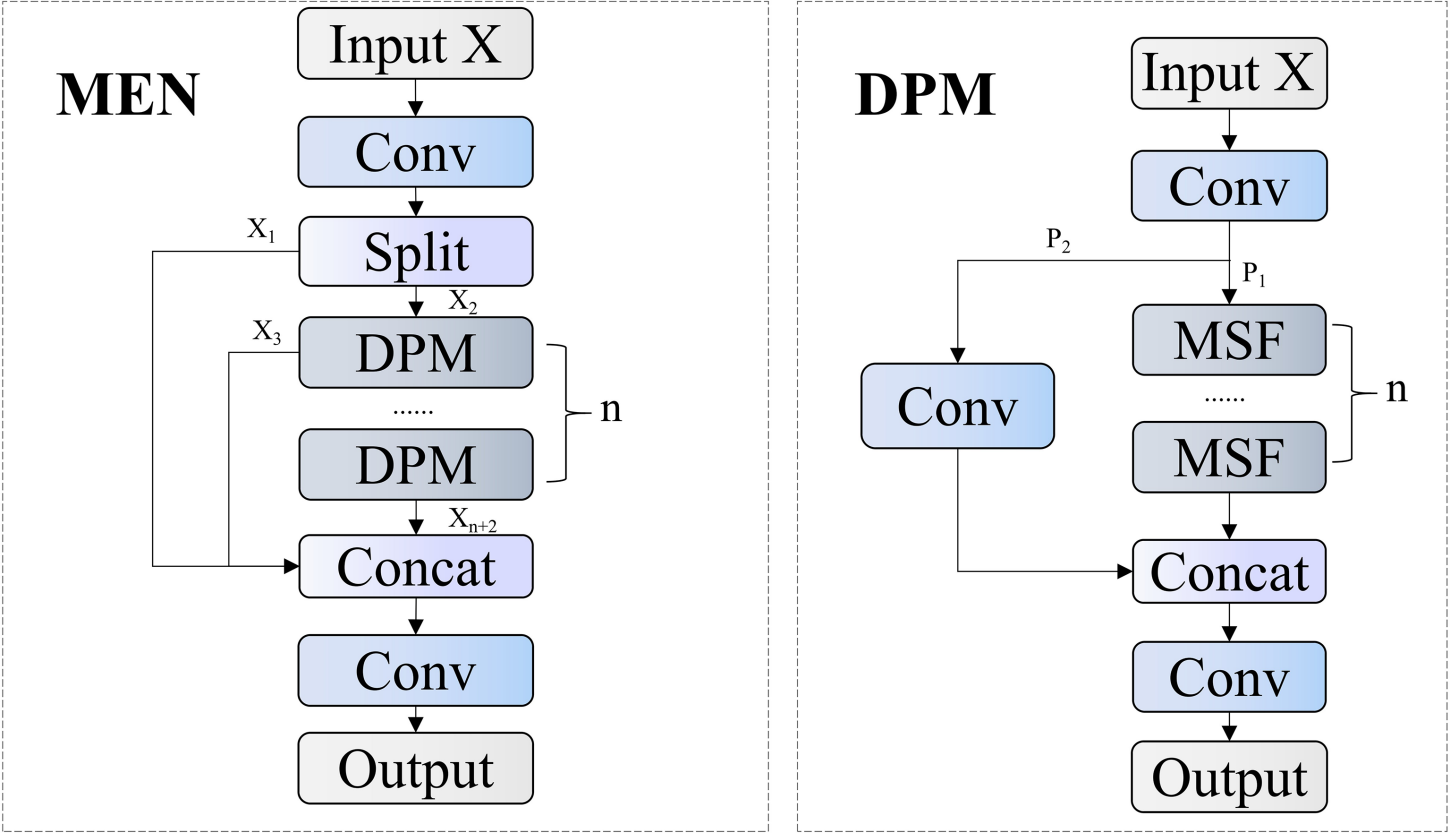
MEN model workflow.

Conventional convolution operations often neglect fine morphological features of small pests, reducing detection precision. Moreover, edge information progressively attenuates as network depth increases. To address these issues, this study proposes the Morphological Edge Network (MEN) module for restructuring the backbone network architecture, as shown in **Figure 6**. This module significantly enhances rice pest detection accuracy and environmental robustness through the organic combination of multi-scale feature representation and edge information enhancement mechanisms.

The MEN module is innovatively designed based on the Cross Stage Partial (CSP) structure, organically combining feature extraction with information enhancement mechanisms. This module receives an input feature map $X\in {\mathbb{R}}^{H\times W\times C_{in}}$ and generates an enhanced feature map $Y\in {\mathbb{R}}^{H\times W\times C_{out}}$ through complex nonlinear transformations. The overall transformation process can be described by the following unified expression, as shown in Equation 1:

(1) $$\begin{array}{c} Y={ℱ}_{fusion}\left(\text{Concat}\left[X_{1},X_{2},{\left\{{ℳ}_{\theta_{i}}\left(X_{2}\right)\right\}}_{i=1}^{n}\right]\right) \end{array}$$

In this formula, $X_{1}$ and $X_{2}$ are two parts of features split along the channel dimension after the input feature X undergoes 1×1 convolution, ${ℳ}_{\theta_{i}}$ represents the MPM transformation function with parameters $\theta_{i}$, n denotes the number of enhancement units in the module, and ${ℱ}_{fusion}$ is the final feature fusion function, typically implemented by 1×1 convolution.

Within the module, DPM serves as the basic unit implementing cross-feature enhancement. This unit adopts a dual-path design, significantly enhancing the model’s feature expression capability. Assuming an input feature X, the output feature Z of the unit can be expressed as shown in Equation 2:

(2) $$\begin{array}{c} Z=G\left(\text{Concat}\left[ℰ\left(\text{one }P\left(X\right)\right),Q\left(X\right)\right]\right) \end{array}$$

where P and Q represent two parallel 1×1 convolution paths, ℰ denotes a tandem sequence composed of MSF units, and G is the fusion function integrating features from both paths. Compared to traditional CSP structures, our design introduces more complex edge-aware mechanisms in the feature extraction path, substantially enhancing the model’s detection capability for pest contours.

**Figure 7:**
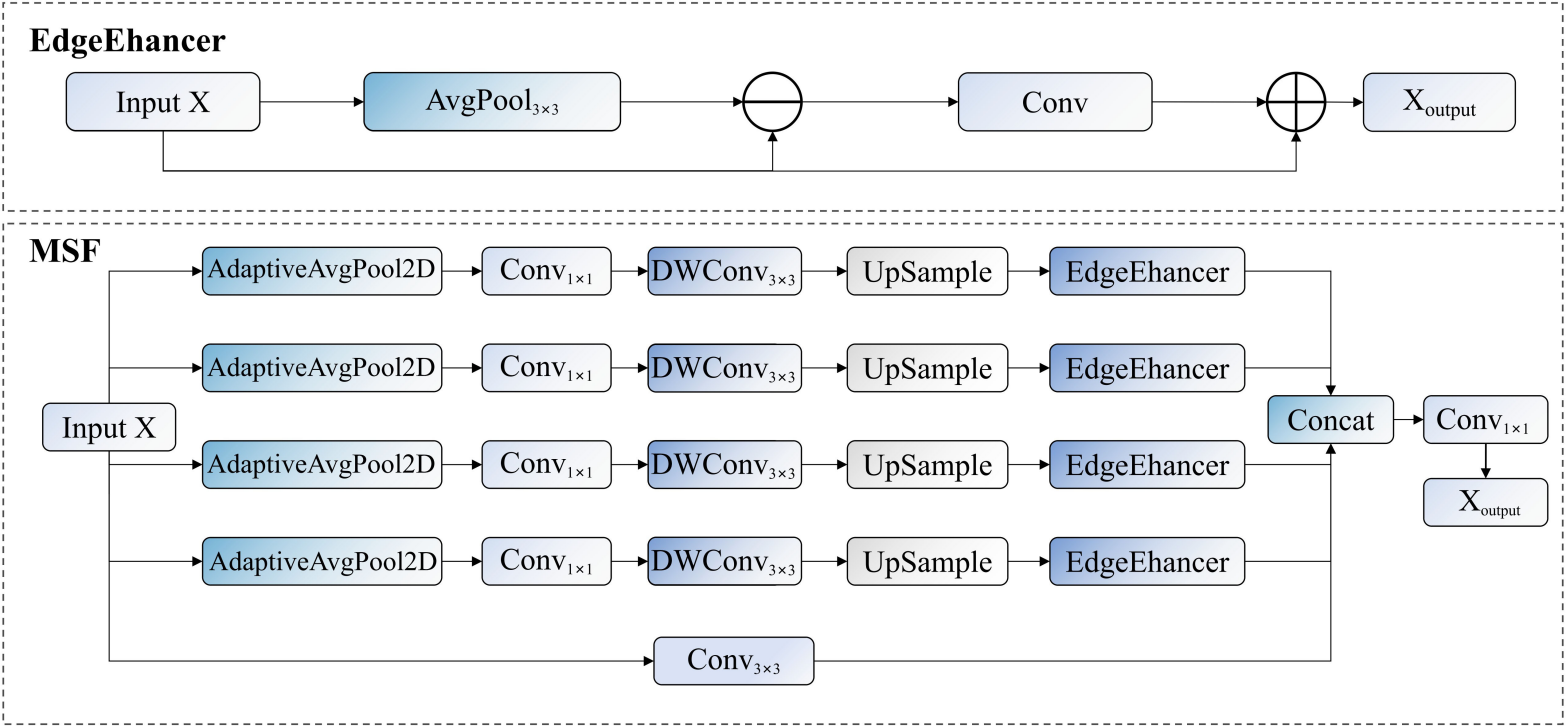
MSF module workflow, where ⊕ represents element-wise addition operation, ⊖ represents element-wise subtraction operation.

MSF focuses on achieving multi-scale feature acquisition and edge information enhancement, with its structure shown in **Figure 7**. This module adopts a multi-branch parallel architecture, simultaneously processing feature expressions of different abstraction levels. Given an input feature $X\in {\mathbb{R}}^{H\times W\times C}$, the mathematical expression of the entire module can be unified as shown in Equation 3:

(3) $$\begin{array}{c} F_{out}=\Phi \left(\text{Concat}\left[ℒ\left(X\right),{\left\{T_{s}\left(A_{s}\left(X\right)\right)\right\}}_{s\in S}\right]\right) \end{array}$$

In this expression, ℒ represents the local feature extraction function, implemented through 3×3 convolution; $S=\left\{s_{1},s_{2},\ldots ,s_{k}\right\}$ denotes a set of predefined scale parameters (e.g., {3,6,9,12}); $A_{s}$ is the adaptive pooling and feature transformation function at scale s; $T_{s}$ represents the transformation function including upsampling and edge enhancement; and Φ is the final feature fusion function.

In the multi-scale feature path, the EdgeEnhancer submodule plays a crucial role, designed to strengthen target edge information and enhance the model’s perception of pest contours. Given an input feature X, the processing of EdgeEnhancer can be mathematically formulated as shown in Equation 4:

(4) $$\begin{array}{c} E_{out}=X+X⊙\sigma \left(ℋ\left(X-P_{avg}\left(X\right)\right)\right) \end{array}$$

In this formula, $P_{avg}$ represents the average pooling operation, used to simulate background information of local regions; $X-P_{avg}\left(X\right)$ calculates the difference between the original feature and its smoothed version, explicitly extracting edge information; ℋ is a nonlinear transformation function composed of convolutional layers; σ denotes the Sigmoid activation function, mapping edge features to the [0,1] interval as attention weights; and ⊙ represents the Hadamard product (element-wise multiplication). Through this adaptive attention mechanism, the module can precisely enhance feature responses in pest edge regions while suppressing background noise, significantly improving detection accuracy and robustness.

The MEN module proposed in this study, by parallel integration of adaptive average pooling operations with different receptive fields, constructs a feature extraction path with hierarchical multi-scale representation capabilities, effectively capturing discriminative feature information of pests of different sizes. The EdgeEnhancer design in this module, through efficient extraction and enhancement of edge information, significantly improves the model’s discrimination capability for fine morphological features of pests, demonstrating superior recognition performance and classification accuracy especially for morphologically similar pest species.

### 3.3 BAFE module

**Figure 8:**
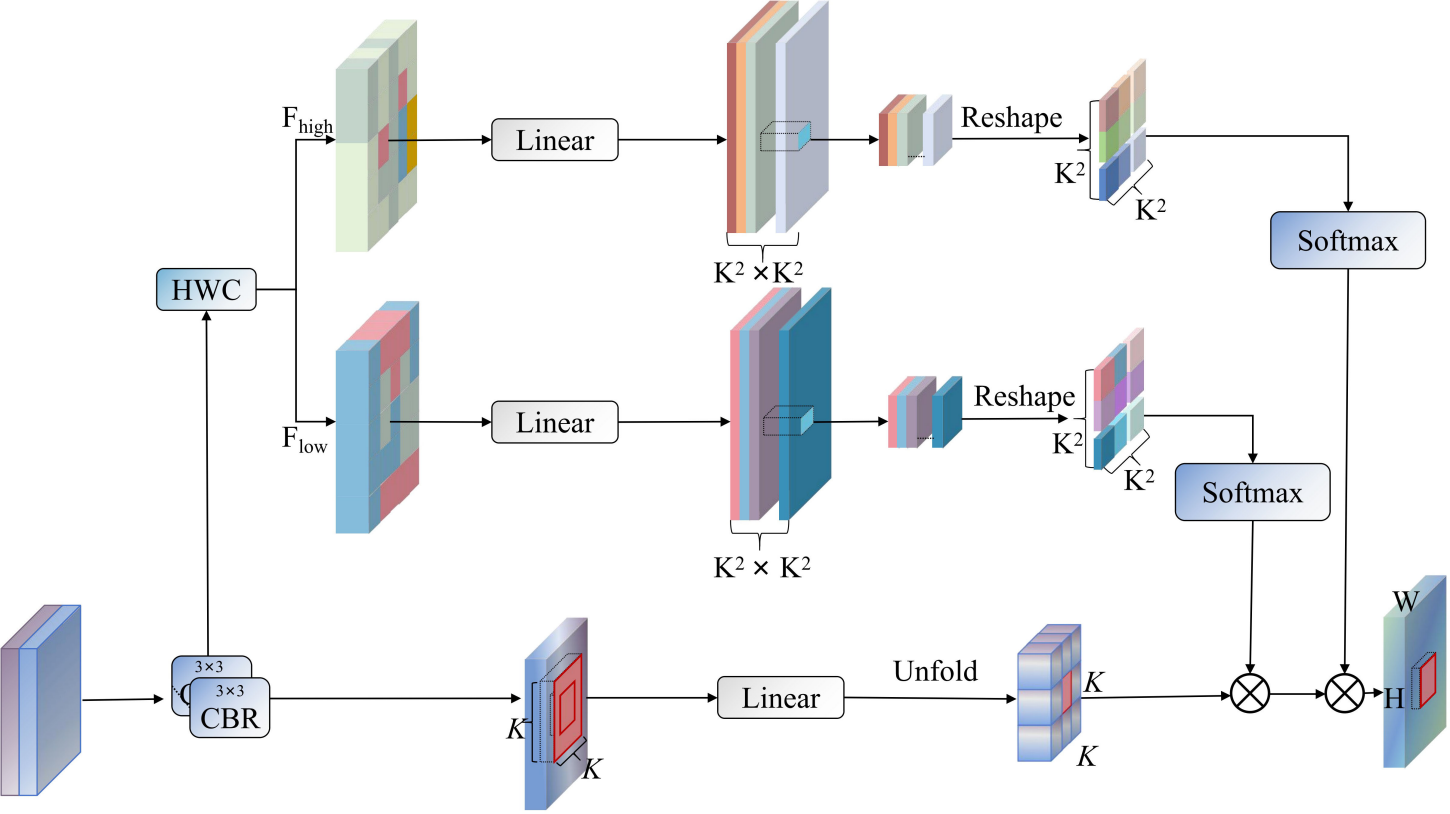
BAFE workflow diagram, where HWC represents HaarWaveletConv, ⊗ represents broadcast element-wise multiplication.

Although the Cross-Stage Partial Spatial Attention (C2PSA) module has achieved remarkable success in object detection, it struggles to discriminate features between pest targets and rice plants, resulting in false positives and false negatives in complex field environments. Single spatial attention mechanisms struggle to capture multi-scale morphological variation features of pests. To address these issues, this paper proposes a novel Bifurcated Attention Feature Enhancement (BAFE), with its structure shown in **Figure 8**. This module significantly improves rice pest detection precision through wavelet transform separation of foreground and background information and the introduction of a dual attention mechanism.

The BAFE module employs frequency domain deconstruction and contrast-driven cascaded attention mechanisms, achieving efficient feature enhancement and aggregation. Given an input feature map $X\in {\mathbb{R}}^{B\times C\times H\times W}$, the module first performs initial feature optimization through an input preprocessing network $\Phi_{in}$, then obtains enhanced features Y through a series of transformation operations. The core component HaarWaveletConv (Su et al., 2024) achieves frequency domain decomposition of features based on discrete Haar wavelet transform principles. This component maps input features to different subbands in the wavelet domain through a set of predefined convolution kernels. Specifically, for input feature X, the wavelet transform process can be expressed as shown in Equation 5:

(5) $$\begin{array}{c} W=H\left(X\right)=\left\{X*\Psi_{d}|d\in \left\{a,h,v,d\right\}\right\} \end{array}$$

where $\Psi_{a}$, $\Psi_{h}$ $\Psi_{v}$, and $\Psi_{d}$ represent Haar filters for approximation (low-pass), horizontal, vertical, and diagonal (high-pass) directions respectively, and ∗ denotes the convolution operation.

Specifically, the HaarWaveletConv employs fixed (non-learnable) Haar wavelet filters initialized according to the standard discrete Haar transform coefficients: ${\text{W}}_{\text{a}}=\frac{1}{2}\left[1,1;1,1\right]$, ${\text{W}}_{\text{h}}=\frac{1}{2}\left[-1,-1;1,1\right]$, ${\text{W}}_{\text{v}}=\frac{1}{2}\left[-1,1;-1,1\right]$, and ${\text{W}}_{\text{d}}=\frac{1}{2}\left[1,-1;-1,1\right]$. The convolution operation uses a stride of 2 and no padding, effectively downsampling the feature map by a factor of 2 while decomposing it into frequency subbands. These filter weights remain frozen during training to preserve the mathematical properties of the Haar wavelet transform.

Among these four subbands, the approximation subband $W_{a}$ contains low-frequency structural information of the image, while the remaining three high-frequency subbands $\left\{W_{h},W_{v},W_{d}\right\}$ capture edge and texture details in different directions. In our implementation, these high-frequency subbands are fused into a single high-frequency feature representation $F_{high}=W_{h}+W_{v}+W_{d}$, which, together with the low-frequency feature $F_{low}=W_{a}$, provides the foundation for subsequent contrast-driven processing. After obtaining frequency domain decomposed features, the module uses a dual attention mechanism to process high-frequency and low-frequency information separately. The foreground attention stage first constructs a spatially sensitive attention map based on high-frequency features. Given the deformed feature $V\in {\mathbb{R}}^{B\times N_{h}\times L\times k^{2}\times d_{h}}$ foreground attention calculation can be represented as shown in Equation 6:

(6) $$\begin{array}{c} A_{fg}=softmax\left(\frac{Q_{fg}\left({\tilde{F}}_{high}\right)\cdot K_{fg}{\left({\tilde{F}}_{high}\right)}^{⊤}}{\sqrt{d_{h}}}+B\right)\in {\mathbb{R}}^{B\times N_{h}\times L\times k^{2}\times k^{2}} \end{array}$$

where ${\tilde{F}}_{high}$ is the high-frequency feature after pooling downsampling, $Q_{fg}$ and $K_{fg}$ are query and key transformation functions respectively, B is positional encoding, $N_{h}$ is the number of attention heads, L is the number of feature spatial positions, k is the convolution kernel size, and $d_{h}$ is the feature dimension of each head. This attention mechanism enhances perception of edges and textures of small pests by considering local spatial dependencies. After applying attention weights to value feature V, an enhanced feature representation is obtained as shown in Equation 7:

(7) $$\begin{array}{c} X_{fg}={ℱ}_{proj}\left(\sum_{i=1}^{k^{2}}A_{fg}\left[:,:,:,:,i\right]⊙V\left[:,:,:,i,:\right]\right) \end{array}$$

where ⊙ represents broadcast element-wise multiplication, and ${ℱ}_{proj}$ is projection transformation. Similarly, the background attention stage utilizes low-frequency features to guide further enhancement of the first stage output, forming the final feature representation. This process can be formalized as shown in Equations 8, 9:

(8) $$\begin{array}{c} A_{bg}=softmax\left(\frac{Q_{bg}\left({\tilde{F}}_{low}\right)\cdot K_{bg}{\left({\tilde{F}}_{low}\right)}^{⊤}}{\sqrt{d_{h}}}+B\right) \end{array}$$

(9) $$\begin{array}{c} Y=\Phi_{out}\left({ℱ}_{proj}\left(\sum_{i=1}^{k^{2}}A_{bg}\left[:,:,:,:,i\right]⊙V^{\prime }\left[:,:,:,i,:\right]\right)\right) \end{array}$$

This cascaded contrast attention design enables the module to progressively refine feature representations under the guidance of different frequency domain information, particularly suitable for capturing subtle differences of rice pests in complex backgrounds. Notably, by using sliding window (unfold) operations and position-sensitive attention mapping, the module can efficiently process spatial dependencies, enhancing representation capability for small targets and pests with complex morphologies.

The BAFE module proposed in this paper achieves adaptive decomposition of features through wavelet transform, effectively separating low-frequency background information from high-frequency foreground information, resolving the foreground-background feature confusion problem in traditional detection networks and enabling the model to focus more precisely on pest targets. It also designed a dual attention mechanism, processing foreground and background information separately and forming a contrast enhancement effect through a stacked approach, significantly enhancing the model’s recognition capacity for morphologically variable and minuscule rice pests.

### 3.4 EM-BFPN

**Figure 9:**
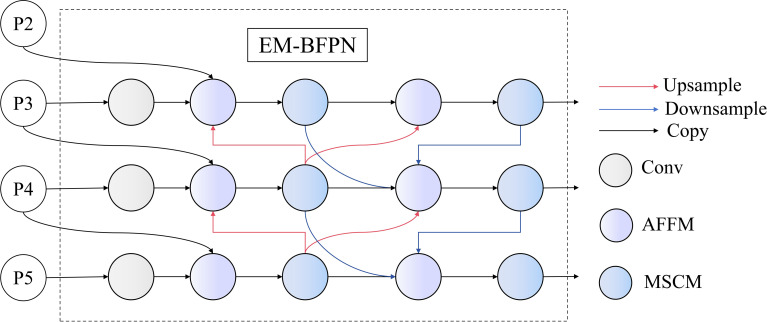
EM-BFPN structure.

Traditional neck networks exhibit evident limitations in feature fusion, typically employing simple concatenation or weighted summation without adequately considering inter-layer correlation and complementarity. This results in poor detection performance for small, dense, and morphologically variable targets such as rice pests. To address these issues, this paper proposes an Enhanced Multi-scale Bidirectional Feature Pyramid Network (EM-BFPN), with its structure shown in **Figure 9**. This network achieves efficient information interaction and complementary information mining between different feature layers through the design of an Adaptive Feature Fusion Mechanism (AFFM) and Multi-scale Convolution Module (MSCM).

The workflow of EM-BFPN can be divided into three stages: feature preprocessing, multi-scale bidirectional feature fusion, and feature enhancement. In the feature preprocessing stage, P3, P4, and P5 feature maps from the backbone network are first unified in channels through 1×1 convolution to reduce computational complexity and improve feature fusion efficiency. In the multi-scale bidirectional feature fusion stage, EM-BFPN implements bidirectional feature transmission from top-down and bottom-up, forming a closed-loop feedback mechanism. The feature fusion process is implemented by the AFFM mechanism, which performs weighted fusion of features from different sources through learnable weight coefficients. The feature enhancement stage employs the innovative MSCM module, which can extract features under multiple receptive fields, effectively enhancing the model’s adaptability to pests of different scales. Finally, P3, P4, and P5 features enhanced through multi-level feature interaction are sent to the detection head for target classification and localization.

The AFFM module in EM-BFPN is a key component for achieving efficient feature fusion. Unlike simple feature concatenation or summation, this module adaptively adjusts the contribution of different features through learnable weight parameters.

For a set of input features $\left\{F_{1},F_{2},\ldots ,F_{n}\right\}$, a learnable weight parameter $w_{i}$ is first defined for each input feature. Then, {w1',w2',…,wn'} are obtained through ReLU activation, ensuring that each input feature’s contribution in the fusion process is non-negative, avoiding mutual cancellation between features. Finally, after weight normalization, the weighted sum of input features is output as shown in Equation 10:

(10) Ffused=∑i=1nwi'·Fi∑j=1nwj'+ϵ,i∈{1,2,…,n}

where $\epsilon =10^{-4}$ is a small constant to prevent division by zero. Compared to fixed-weight fusion methods, this learning-based fusion mechanism can dynamically adjust the importance of different features, adapting to different detection scenarios. Through end-to-end training, weight parameters can be automatically optimized according to the loss function without manual adjustment, maintaining computational efficiency.

**Figure 10:**
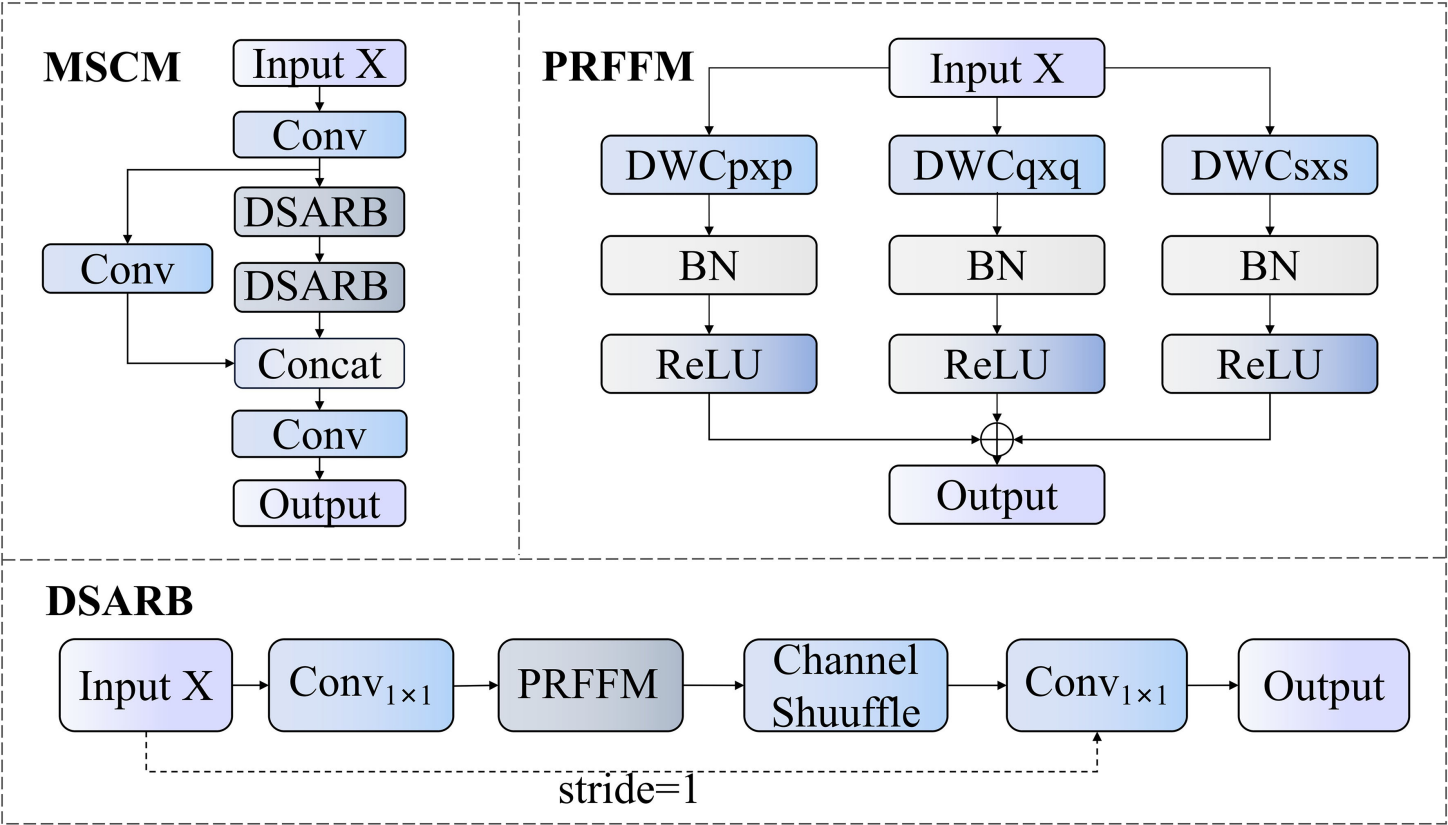
MSCM internal structure.

The MSCM module integrates the advantages of cross-stage partial networks and multi-scale convolution, effectively extracting and processing multi-scale feature information, with its structure shown in **Figure 10**. Given an input feature $X\in {\mathbb{R}}^{C_{1}\times H\times W}$, the forward propagation process of MSCM can be represented as shown in Equation 11:

(11) $$\begin{array}{c} \Phi_{CSP}\left(X\right)={\text{Conv}}_{1\times 1}\left(\text{Concat}\left[\phi_{1}\left({\text{Conv}}_{1\times 1}\left(X\right)\right),\phi_{2}\left(\prod_{i=1}^{n}DSARB_{i}\left(\phi_{3}\left({\text{Conv}}_{1\times 1}\left(X\right)\right)\right)\right)\right]\right) \end{array}$$

where $\phi_{1},\phi_{3}:{\mathbb{R}}^{C_{1}}\to {\mathbb{R}}^{C_{1}\cdot e/2}$ and $\phi_{2}:{\mathbb{R}}^{C_{1}\cdot e/2}\to {\mathbb{R}}^{C_{1}\cdot e/2}$ represent feature transformation functions respectively, e is the expansion coefficient, Concat[·] denotes feature concatenation operation along the channel dimension, and $\prod_{i=1}^{n}DSARB_{i}$ represents the combined operation of multiple cascaded Dynamic Scale-Adaptive Residual Blocks (DSARB).

DSARB employs parallel multi-scale depth-separable convolution to enhance feature extraction capability. The complete computation process of DSARB can be represented as shown in Equation 12:

(12) $$\begin{array}{c} Y=\{\begin{array}{cc} X+{ℱ}_{proj}\left(\Psi {(}_{ℳ}\left({ℱ}_{\text{exp}}\left(X\right)\right))\right), & \text{if}\;s=1\\ {ℱ}_{proj}\left(\Psi {(}_{ℳ}\left({ℱ}_{\text{exp}}\left(X\right)\right))\right), & \text{if}\;s=2 \end{array} \end{array}$$

where ${ℱ}_{\text{exp}}:{\mathbb{R}}^{C_{in}\times H\times W}\to {\mathbb{R}}^{e\cdot C_{in}\times H\times W}$ is pointwise convolution for channel expansion, e is the expansion coefficient; _*ℳ*_ is the multi-scale feature aggregation function, representing the feature aggregation result after the Parallel Receptive Field Fusion Module (PRFFM); Ψ represents the Channel Shuffle operation (Zhang and Yang, 2021); ${ℱ}_{proj}:{\mathbb{R}}^{e\cdot C_{in}\times H\times W}\to {\mathbb{R}}^{C_{out}\times H\times W}$ is pointwise convolution for channel projection; ${ℱ}_{res}:{\mathbb{R}}^{C_{in}\times H\times W}\to {\mathbb{R}}^{C_{out}\times H\times W}$ is 1×1 convolution for residual connection; and s represents the convolution stride.

The PRFFM module is the core component of DSARB, implementing parallel multi-scale depth-separable convolution. Given an input feature $X\in {\mathbb{R}}^{C\times H\times W}$ and a set of convolution kernel sizes $K=\left\{k_{1},k_{2},\ldots ,k_{m}\right\}$, the output of PRFFM can be represented as shown in Equation 13:

(13) $$\begin{array}{c} {ℱ}_{msdc}\left(X\right)=\left\{Y_{1},Y_{2},\ldots ,Y_{m}\right\},\;\;Y_{i}={ℱ}_{DW}^{k_{i}}\left(X\right)=\sigma \left(ℬN\left(\sum_{c=1}^{C}W_{c}^{k_{i}}*X_{c}\right)\right) \end{array}$$

where ${ℱ}_{DW}^{k_{i}}:{\mathbb{R}}^{C\times H\times W}\to {\mathbb{R}}^{C\times H'\times W'}$ represents the depth-separable convolution operation with kernel size $k_{i}$: where $W_{c}^{k_{i}}\in {\mathbb{R}}^{1\times k_{i}\times k_{i}}$ is the convolution kernel weight of the c-th channel, * represents the two-dimensional convolution operation, ℬN represents batch normalization, and σ represents the ReLU nonlinear activation function.

The EM-BFPN neck network structure proposed in this paper has been specially optimized for rice pest detection scenarios. Through multi-path feature flow and iterative feature fusion, it significantly enhances feature reuse efficiency and multi-scale information interaction, addressing the limitations of traditional FPN in processing rice pest targets with significant scale variations. By adopting the AFFM fusion mechanism, the network can adaptively adjust the importance of features at different scales, enhancing selective extraction of key information and avoiding information redundancy and noise interference problems caused by traditional simple fusion methods.

### 3.5 SCAU upsampling submodule

**Figure 11:**
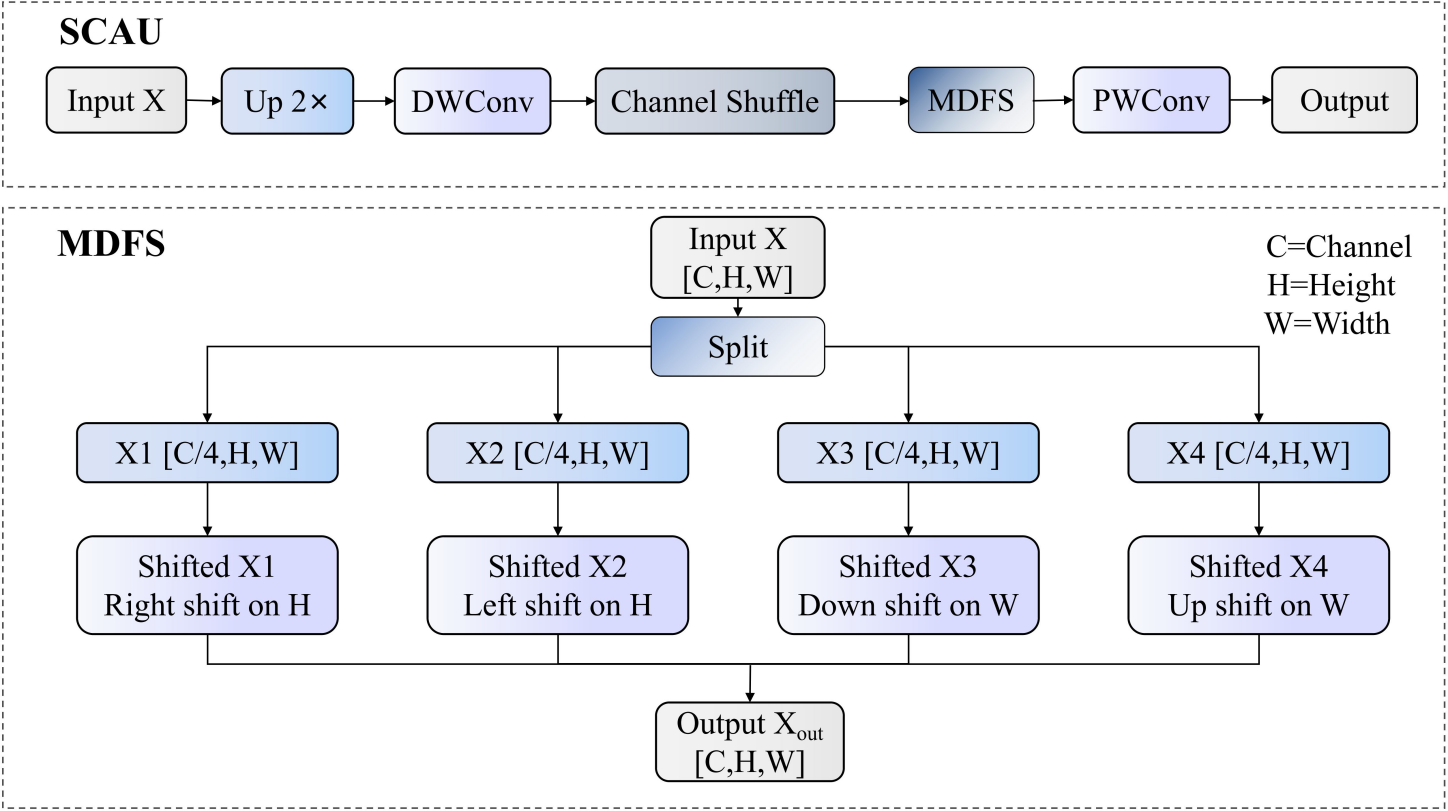
SCAU module structure.

Traditional upsampling modules typically employ bilinear or nearest-neighbor interpolation. While computationally efficient, these methods often cause information loss and spatial blurring during feature map reconstruction. Particularly in rice pest detection tasks, where pest targets are typically small in volume, morphologically similar, and in complex backgrounds, such information loss significantly affects detection precision. To address these issues, this paper proposes an upsampling module called Spatial-Channel Augmented Upsampling (SCAU), as shown in **Figure 11**. This module effectively enhances the model’s capability to detect small-sized rice pests and distinguish between similar pest categories by employing a Channel Shuffle mechanism and innovative Multi-Directional Feature Shifting (MDFS) units.

The workflow of the SCAU module primarily includes four stages: upsampling, Channel Shuffle, Multi-Directional Feature Shifting, and pointwise convolution. The input feature map X is processed through an upsampling layer combined with depth-separable convolution (DWConv), then processed through the Channel Shuffle mechanism to obtain X". This operation ensures that channel information from different groups can be thoroughly mixed, enhancing the diversity of feature expression. After channel shuffling, the feature map enters the MDFS unit for spatial shift mixing, followed by further fusion of channel information through pointwise convolution (PWConv) to obtain the final output map.

The MDFS unit is the core innovation of the SCAU module, aimed at enhancing the spatial context perception capability of feature maps without increasing parameter count. For an input feature map $X\in {\mathbb{R}}^{C\times H\times W}$, the MDFS unit first evenly divides it along the channel dimension into four sub-feature maps, obtaining $X_{1},X_{2},X_{3},X_{4}$. Each sub-feature map $X_{i}\in {\mathbb{R}}^{\frac{C}{4}\times H\times W}$, i∈{1,2,3,4}, contains one-quarter of the channels of the input feature map.

After division, different directions and magnitudes of circular shift operations are applied to these four sub-feature maps, as shown in Equations 14–17:

(14) $$\begin{array}{c} X_{1}^{'}=T_{H,s}\left(X_{1}\right)=Roll\left(X_{1},shift=s,dims=2\right) \end{array}$$

(15) $$\begin{array}{c} X_{2}^{'}=T_{H,-s}\left(X_{2}\right)=Roll\left(X_{2},shift=-s,dims=2\right) \end{array}$$

(16) $$\begin{array}{c} X_{3}^{'}=T_{W,s}\left(X_{3}\right)=Roll\left(X_{3},shift=s,dims=3\right) \end{array}$$

(17) $$\begin{array}{c} X_{4}^{'}=T_{W,-s}\left(X_{4}\right)=Roll\left(X_{4},shift=-s,dims=3\right) \end{array}$$

In these formulas, Roll represents the circular shift operation, $T_{H,s}$ and $T_{W,s}$ represent shift transformation functions in height and width dimensions respectively, and s is the shift amount. Specifically, dims=2 indicates shifting in the height dimension, and dims=3 indicates shifting in the width dimension. Positive shift values indicate downward or rightward movement of features, while negative shift values indicate upward or leftward movement. After the shift operations, the four sub-feature maps are reconnected along the channel dimension, as shown in Equation 18:

(18) Xout=Concat([X1',X2',X3',X4'],dim=1)∈ℝC×H×W

where Concat represents the tensor concatenation operation, and dim=1 specifies concatenation along the channel dimension. The final output feature map $X_{out}$ has the same shape as the input feature map X but contains enhanced spatial context information.

The SCAU module proposed in this paper addresses small target recognition and similar category discrimination problems in rice pest detection tasks through upsampling combined with depth-separable convolution, channel shuffling mechanism, and spatial shift mixing strategy, effectively enhancing feature map expression capability without significantly increasing computational complexity. Compared to traditional upsampling methods, the SCAU module reduces computation while preserving spatial information by employing depth-separable convolution. The module adopts a channel shuffling mechanism to promote interactive fusion of different channel features, enhancing the model’s representation capability.

## 4 Experiments

### 4.1 Experimental environment and hyperparameter settings

The hardware configuration for this experiment comprised computing nodes and graphics processors, with specific environmental settings and experimental parameters detailed in **Tables 1**, **2**. The central processing unit utilized was a 12th Gen Intel(R) Core(TM)i7-12650H, while the graphics processor employed was an NVIDIA GeForce RTX 4060. The software environment was deployed on the Windows 11 operating system, with a programming environment based on Python 3.9 and the PyTorch 11.7 deep learning framework. Specific parameters for model training were configured as follows: iteration period (epoch) was set to 250, batch size to 16, and the optimization algorithm utilized was Stochastic Gradient Descent (SGD) with an initial learning rate of 0.01 and a momentum factor of 0.937, with other parameters adopting default values. Input data dimensions were uniformly adjusted to 640×640 pixel resolution through standardized preprocessing.

**Table 1:** Experimental platform.

Platform	Name
CPU	12th Gen Intel(R) Core(TM)i7-12650H
GPU	NVIDIA GeForce RTX 4060
The operating system	Windows 11
Programming language	Python 3.9
Deep learning framework	Pytorch 11.7

**Table 2:** Some experimental details of each framework.

Name	Value
epoch	250
Batch size	16
optimizer	SGD
Initial learning rate	0.01
momentum	0.937
Image size	640×640

### 4.2 Ablation experiments

To verify the effectiveness of the proposed BEAM-YOLO algorithm in rice pest detection tasks, we conducted ablation experiments on four core innovations. Here, A represents the MEN module, B represents the BAFE module, C represents the EM-BFPN module, and D represents the SCAU module. Starting from the baseline model, each innovative module was progressively added to evaluate their independent and combined contributions to detection performance. The ablation experiment results are shown in **Table 3**.

**Table 3:** Ablation experiment results.

Method	mAP@50	mAP@50-95	Recall	Precision	FLOPS	Params
BaseLine	83.3 ± 0.5%	69.7 ± 1.1%	85.1 ± 0.3%	74.9 ± 0.6%	6.3G	2.5M
A	83.7 ± 0.6%	69.9 ± 0.8%	80.5 ± 0.7%	78.3 ± 0.8%	6.5G	2.56M
B	83.5 ± 0.5%	69.8 ± 1.0%	86.2 ± 0.6%	76.1 ± 0.6%	8.6G	5.20M
C	84.1 ± 0.6%	70.3 ± 1.0%	82.7 ± 0.6%	76.8 ± 0.7%	6.8G	2.14M
D	83.9 ± 0.5%	70.0 ± 0.7%	84.2 ± 0.8%	75.7 ± 0.6%	6.9G	2.70M
A+C	85.2 ± 0.3%	71.5 ± 0.9%	82.3 ± 0.5%	79.1 ± 1.0%	6.6G	2.09M
A+D	84.5 ± 0.5%	70.8 ± 0.5%	82.7 ± 0.6%	79.5 ± 0.8%	6.9G	2.65M
B+C	85.7 ± 0.5%	71.7 ± 0.9%	87.2 ± 0.3%	77.5 ± 0.6%	8.7G	4.72M
B+D	85.3 ± 0.3%	71.4 ± 0.8%	87.5 ± 0.5%	77.2 ± 0.7%	9.0G	5.28M
A+B+C	86.0 ± 0.3%	72.0 ± 0.8%	81.5 ± 0.5%	78.0 ± 0.8%	8.7 G	4.67M
A+B+D	85.9 ± 0.4%	72.3 ± 1.0%	85.4 ± 0.5%	79.3 ± 0.8%	9.0G	5.23M
B+C+D	86.2 ± 0.6%	72.4 ± 0.7%	86.8 ± 0.6%	78.4 ± 0.6%	8.8G	4.73M
A+B+C+D	86.6 ± 0.5%	72.7 ± 0.9%	83.3 ± 0.5%	78.8 ± 0.7%	8.8 G	4.68M

**Figure 12:**
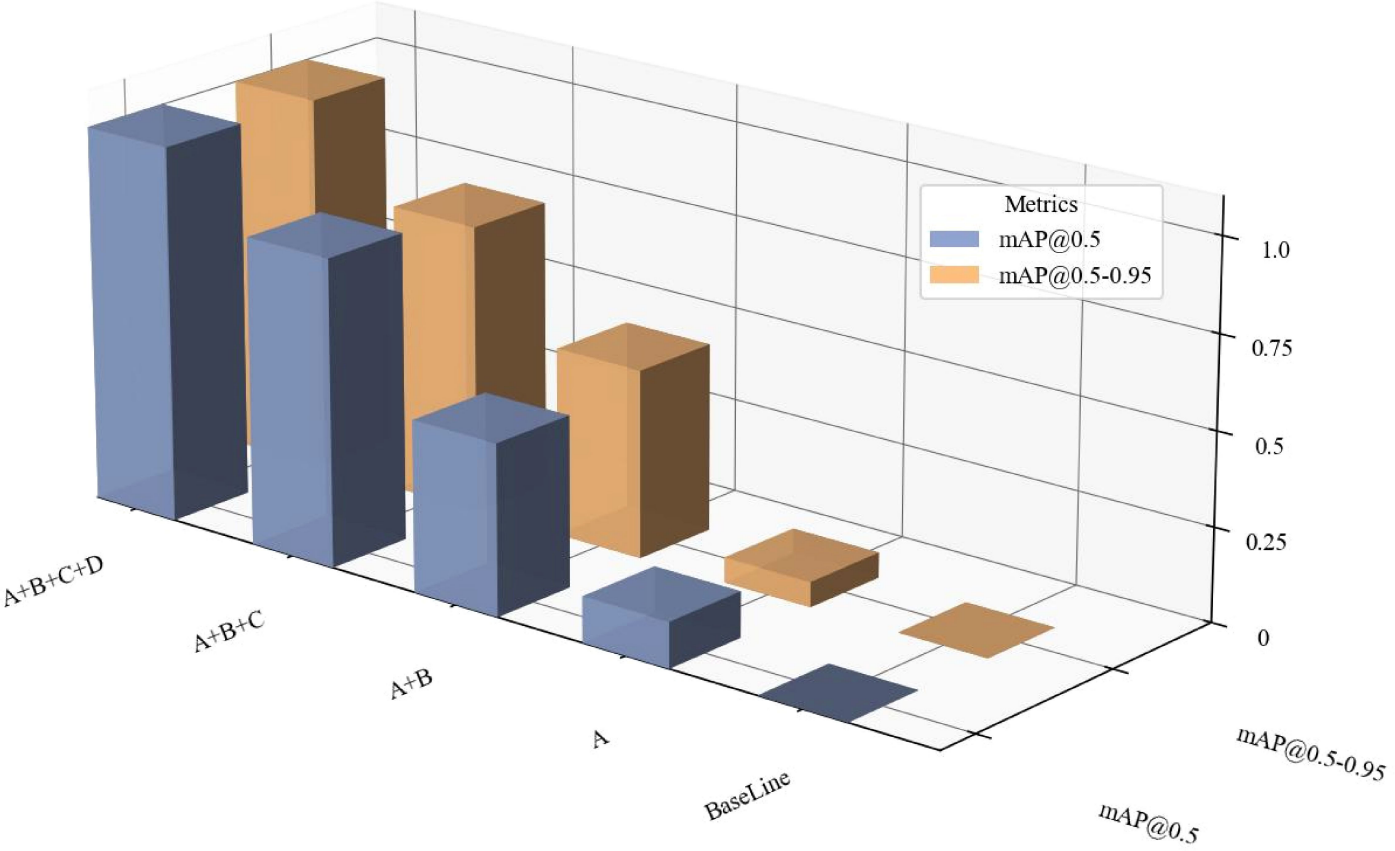
Ablation parameter bar chart.

The experimental results clearly demonstrate the significant performance improvement brought by the proposed innovative modules. When all four modules were combined, our proposed BEAM-YOLO model achieved 86.6% mAP@50, a 3.3% improvement over the baseline, and reached 72.7% under the more stringent mAP@50–95 evaluation criterion, a 3.0% improvement. Notably, the combination of the MEN module and EM-BFPN achieved significant performance improvement while maintaining relatively low computational complexity and parameter count. The BAFE module showed exceptional performance in improving Recall, enabling the model to better detect difficult-to-recognize pest targets. **Figure 12** illustrates the ablation experiment results for different network component combinations through a three-dimensional bar chart, intuitively presenting the performance of each configuration on two key metrics: mAP@50 and mAP@50-95. A clear progressive improvement trend in model performance can be observed as components are accumulated.

### 4.3 Comparative experiments

#### 4.3.1 Comparison of different backbones

To verify the effectiveness of the proposed improved YOLOv11 backbone network in rice pest detection tasks, we conducted comprehensive comparative experiments. Five mainstream backbone networks in the current object detection field were selected, including Resnet18 (Odusami et al., 2021), Repvit (Wang et al., 2024a), EfficientViT (Liu et al., 2023), and Unireplknet (Ding et al., 2024), for performance comparison with our improved network that integrates the MEN and BAFE modules. Through these comparative experiments, we aimed to evaluate the enhancement effect of the proposed multi-scale edge enhancement and contrastive wavelet attention mechanism on rice pest detection performance.

The experimental results show that our proposed backbone network achieved significant advantages across all metrics. As shown in **Table 4**, our improved backbone network model demonstrated optimal performance, with mAP@50 improved by 1.5%, mAP@50–95 by 1.5%, recall rate by 1.6%, and precision by 3.7% compared to the baseline YOLOv11 model. Meanwhile, our model used only 5.1M parameters with a computational load of 8.6GG, reducing parameter count by 60.8% and computational load by 74.4% compared to Resnet18, and reducing parameter count by 20.3% and computational load by 49.4% compared to Repvit. These results fully validate the effectiveness of the backbone network reconstituted with the MEN module and BAFE module, where the MEN module significantly enhanced detection capability for pests of different sizes through multi-scale feature extraction and edge enhancement, while the BAFE module improved recognition precision for morphologically variable and tiny rice pests through its dual attention mechanism.

**Table 4:** Comparison experiment results of different backbones.

Method	mAP@50	mAP@50-95	Recall	Precision	FLOPS	Params
BaseLine	83.3 ± 0.5%	69.7 ± 1.1%	85.1 ± 0.3%	74.9 ± 0.6%	6.3G	2.5M
Resnet18	82.6 ± 0.7%	68.3 ± 0.7%	86.1 ± 0.4%	68.4 ± 1.0%	33.6G	13.0M
Repvit	82.7 ± 0.6%	70.7 ± 0.5%	77.2 ± 0.7%	76.1 ± 0.6%	17.0G	6.4M
EfficientViT	81.9 ± 0.5%	68.0 ± 0.9%	82.8 ± 0.8%	76.6 ± 0.8%	7.9G	3.7M
Unireplknet	79.6 ± 0.8%	65.2 ± 0.6%	86.0 ± 0.3%	66.9 ± 0.6%	14.1G	5.8M
Ours	84.8 ± 0.6%	71.2 ± 0.9%	86.7 ± 0.6%	78.6 ± 0.6%	8.6 G	5.1M

**Figure 13:**
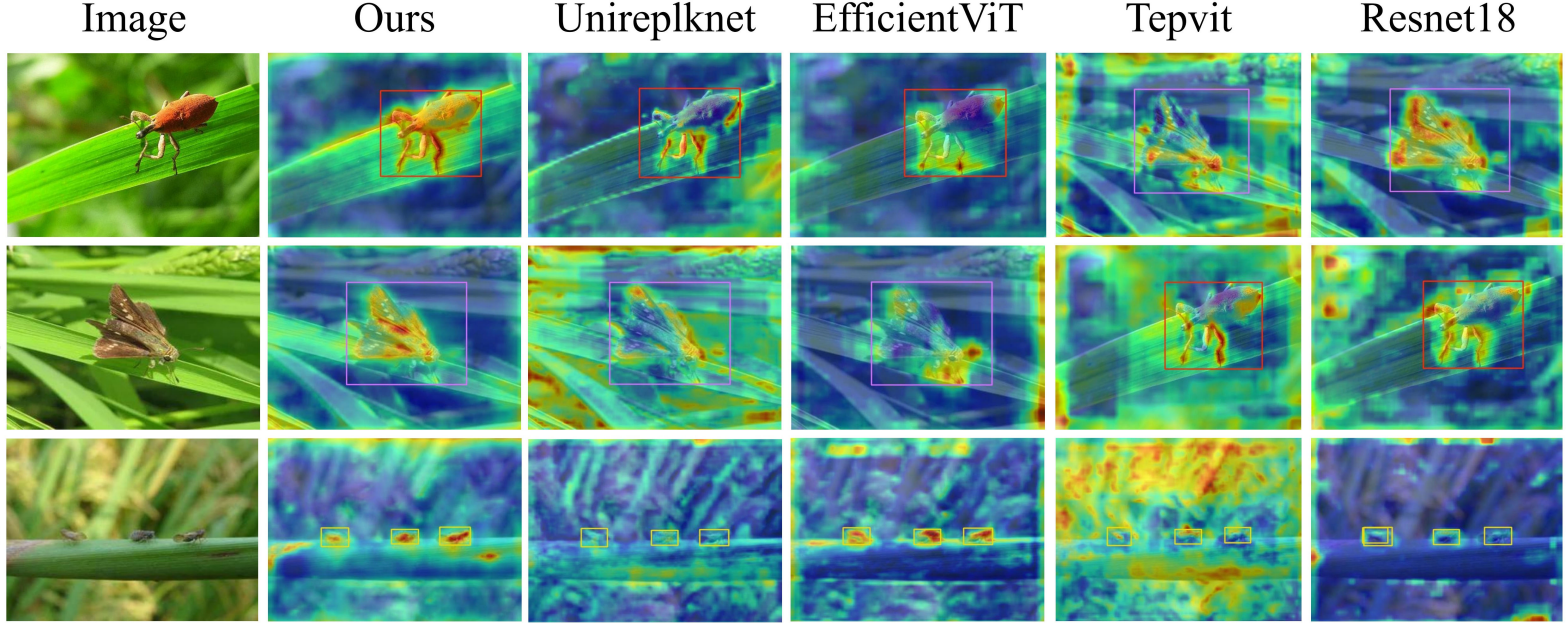
Heatmap visualization of different backbone networks.

To further verify the feature extraction performance of our proposed model, we generated feature response heatmaps for various backbone networks on the rice pest dataset, as shown in **Figure 13**. The visualization results quantitatively demonstrate the significant advantage of our proposed backbone network integrating MEN and BAFE modules in target feature extraction. Compared to the control group networks, our proposed network architecture exhibited higher feature selectivity, more precise localization of pest target regions, higher intensity of concentrated activation, and clearer boundary definition in the heatmaps. This result further confirms that the multi-scale edge enhancement mechanism of the proposed MEN module and the foreground-background contrastive attention mechanism of the BAFE module can work synergistically to effectively improve the detection performance of small rice pest targets.

#### 4.3.2 Comparison of different Neck-FPNs

To verify the effectiveness of our proposed neck network in rice pest detection tasks, we compared five mainstream neck network architectures, including Slim-neck (Li H. et al., 2022), MAFPN (Zhang et al., 2023), GFPN (Zhao et al., 2021), EfficientRepBiPAN (Ye et al., 2024), and Bifpn (Chen et al., 2021), with the experimental results shown in **Table 5**. All comparative experiments were conducted based on the same backbone network and detection head, ensuring fair comparison of the performance differences between various neck networks. The experiments focused on evaluating various model metrics, comprehensively analyzing the advantages and limitations of different neck networks in detecting small targets and multi-morphological rice pests.

**Table 5:** Experiment results of different neck networks.

Method	mAP@50	mAP@50-95	Recall	Precision	FLOPS	Params
BaseLine	83.3 ± 0.5%	69.7 ± 1.1%	85.1 ± 0.3%	74.9 ± 0.6%	6.3G	2.5M
Slim-neck	81.6 ± 0.5%	68.0 ± 0.9%	86.0 ± 0.3%	70.0 ± 0.7%	5.9G	2.5M
MAFPN	77.9 ± 1.0%	65.3 ± 0.7%	78.5 ± 0.9%	69.7 ± 1.1%	7.1G	2.6M
GFPN	80.5 ± 0.7%	67.9 ± 1.0%	81.0 ± 0.5%	72.4 ± 0.7%	8.2G	3.6M
EfficientRepBiPAN	77.5 ± 0.6%	62.9 ± 1.0%	78.8 ± 0.7%	68.0 ± 0.9%	7.8G	3.1M
Bifpn	77.9 ± 1.0%	64.8 ± 0.9%	76.0 ± 0.6%	72.0 ± 0.8%	6.3G	1.9M
Ours	84.6 ± 0.8%	70.5 ± 0.7%	81.6 ± 0.5%	78.4 ± 0.6%	6.7G	2.1M

The experimental results prove that our improved neck network outperforms the comparative methods across all performance metrics. Compared to the baseline YOLOv11 model, our approach improved mAP@50 by 1.3%, mAP@50–95 by 0.8%, and precision by 3.5%. Particularly noteworthy is that our model uses only 2.1M parameters, 16% fewer than YOLOv11 and 41.7% fewer than GFPN. In terms of computational efficiency, our model’s FLOPS is 6.7G, only 6.3% higher than the baseline model, but 18.3% lower than GFPN and 14.1% lower than EfficientRepBiPAN. These results indicate that through the EM-BFPN’s adaptive feature fusion mechanism and SCAU’s multi-directional feature shifting strategy, our neck network improves rice pest detection precision while maintaining relatively low computational complexity, making it particularly suitable for resource-constrained environments in practical applications.

#### 4.3.3 Comparison of different datasets

To verify the generalization capability of the proposed BEAM-YOLO model, we conducted cross-validation experiments based on the public dataset pest-dc2xk (Serasinghe, 2022) from the Roboflow Universe platform. This dataset, created by Aryan Serasinghe, contains 1,003 high-quality rice pest images covering 10 common rice pest categories, including yellow rice borer, rice leaf roller, rice leafhopper, rice water weevil, rice gall midge, and other morphologically diverse pest species. These images were collected under varying environmental conditions, lighting intensities, and shooting angles, providing an ideal testing foundation for evaluating model robustness in complex real-world scenarios. We divided the dataset into training, validation, and test sets at an 8:1:1 ratio and conducted comparative experiments with other mainstream object detection algorithms, with experimental results shown in **Table 6**.

**Table 6:** Comparative experiments across different datasets.

Dataset	Method	mAP@50	mAP@50-95	Recall	Precision
JRICE-PD	YOLOv11	83.3 ± 0.5%	69.7 ± 1.1%	85.1 ± 0.3%	74.9 ± 0.6%
	BEAM-YOLO	86.6 ± 0.5%	72.7 ± 0.9%	83.3 ± 0.5%	78.8 ± 0.7%
pest-dc2xk	YOLOv11	79.1 ± 1.0%	51.2 ± 1.0%	85.7 ± 0.5%	74.2 ± 0.5%
	BEAM-YOLO	82.6 ± 0.7%	53.5 ± 1.0%	84.8 ± 0.6%	78.6 ± 0.6%

The experimental results demonstrate that BEAM-YOLO outperforms the original YOLOv11 across all evaluation metrics, fully validating the effectiveness of our four innovative components. On the challenging pest-dc2xk dataset, BEAM-YOLO also performed excellently, achieving an mAP@50 of 82.6%, a 3.5% improvement over YOLOv11’s 79.1%; precision also increased from 74.2% to 78.6%, a growth of 4.4%. These results confirm that our MEN module effectively enhanced the ability to extract pest edge features, the BAFE module significantly improved foreground-background feature discrimination, the EM-BFPN optimized fusion efficiency of features at different scales, and the SCAU module improved detection precision for small target pests. Particularly in complex agricultural backgrounds, BEAM-YOLO demonstrated stronger recognition capability and environmental adaptability.

#### 4.3.4 Comparison of different models

To verify the effectiveness of the proposed BEAM-YOLO model in rice pest detection tasks, we compared current mainstream object detection algorithms, including Transformer-based RTDETR series, the classic SSD, and YOLO series models, with our BEAM-YOLO model. To ensure fair comparison, all models were retrained from scratch on the JRICE-PD dataset under identical experimental conditions: 250 epochs, 640×640 input resolution, batch size of 16, SGD optimizer with initial learning rate of 0.01, and the same data augmentation pipeline. The experimental results are shown in **Table 7**, comprehensively evaluating the performance of each model in terms of detection precision, recall rate, computational efficiency, and other aspects.

**Table 7:** Comparison experiment results of different models.

Method	Type	mAP@50	mAP@50-95	Recall	Precision	FLOPS	Params
RTDETR-R18 (Zhu and Kong, 2024)	DETR	73.9 ± 0.9%	51.2 ± 1.0%	69.1 ± 0.6%	84.8 ± 0.6%	57.0G	19.9M
RTDETR-R50 (Zhao et al., 2024)	DETR	69.7 ± 1.1%	47.5 ± 1.2%	72.9 ± 0.8%	78.6 ± 0.6%	129.6G	42.0M
RTDETR-L (Jun et al., 2024)	DETR	70.5 ± 0.7%	47.9 ± 0.8%	77.2 ± 0.7%	81.3 ± 0.4%	103.5G	33.0M
SSD (Liu et al., 2016)	One-Stage	82.2 ± 0.8%	64.4 ± 0.7%	77.3 ± 0.6%	82.1 ± 0.8%	61.2G	24.8M
YOLOv3-tiny (Cheng et al., 2021)	One-Stage	81.9 ± 0.5%	64.2 ± 1.1%	83.1%	73.2 ± 0.9	18.9G	12.1M
YOLOv5 (Wu et al., 2021)	One-Stage	80.2 ± 0.7%	65.6 ± 0.9%	76.0 ± 0.6%	75.2 ± 0.5%	7.1G	25.0M
YOLOv6 (Li C. et al., 2022)	One-Stage	74.8%	61.0%	78.3%	67.6%	11.5G	4.1M
YOLOv8 (Sohan et al., 2024)	One-Stage	81.6 ± 0.5%	68.0 ± 0.9%	78.4 ± 0.6%	73.6 ± 0.8%	8.1G	3.0M
YOLOv9 (Wang C-Y. et al., 2024)	One-Stage	84.4 ± 0.6%	72.5 ± 0.8%	83.3 ± 0.5%	76.1 ± 0.6%	87.2G	21.1M
YOLOv10 (Wang et al., 2024b)	One-Stage	81.6 ± 0.5%	69.0 ± 1.1%	83.9 ± 0.5%	72.7 ± 0.9%	6.5G	2.2M
YOLOv11	One-Stage	83.3 ± 0.5%	69.7 ± 1.1%	85.1 ± 0.3%	74.9 ± 0.6%	6.3G	2.5M
BEAM-YOLO	One-Stage	86.6 ± 0.5%	72.7 ± 0.9%	83.3 ± 0.5%	78.8 ± 0.7%	8.8 G	4.6M

All models in **Table 7** were retrained by the authors using the hyperparameters specified in Section 4.1.1. Model-specific configurations are provided in the supplementary YAML files.

The experimental results clearly demonstrate that the proposed BEAM-YOLO model achieved optimal performance in rice pest detection tasks. On the key metric mAP@50, BEAM-YOLO reached a detection precision of 86.6%, significantly outperforming all comparative models. Compared to Transformer-based RTDETR series, BEAM-YOLO’s mAP@50 surpassed RTDETR-R18 by 12.7% while its computational load was only 15.4% of the latter; compared to the traditional SSD model, it improved by 4.4% with a parameter count of only 18.5%. Within the YOLO series, BEAM-YOLO improved by 3.3% over YOLOv11’s 83.3%; on the mAP@50–95 metric reflecting the model’s scale adaptability, BEAM-YOLO reached 72.7%, representing improvements of 3.0% and 8.3% over YOLOv11’s 69.7% and SSD’s 64.4%, respectively. Most significantly, BEAM-YOLO maintained high detection precision while substantially reducing computational burden compared to models with similar performance, with only 8.8G FLOPS and 4.6M parameters, dramatically lower than YOLOv9.

**Figure 14:**
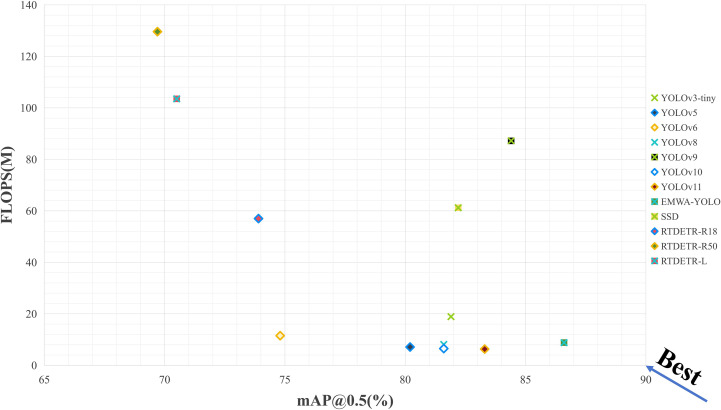
Scatter plot of different model parameters.

Comprehensive analysis indicates that the BEAM-YOLO model effectively captured pest morphological features through the MEN edge enhancement module, successfully separated pest targets from complex agricultural backgrounds through the BAFE module, while the EM-BFPN and SCAU modules enhanced detection capability for multi-scale pests, jointly constructing a rice pest detection algorithm with high precision and strong generalization ability, achieving significant improvement in detection performance while maintaining computational efficiency. **Figure 14** shows a scatter plot of the relationship between detection performance and computational complexity for different YOLO model variants. From the figure, it can be clearly observed that the BEAM-YOLO model exhibits excellent performance in the trade-off between performance and efficiency.

### 4.4 Visualization comparison of detection results

**Figure 15:**
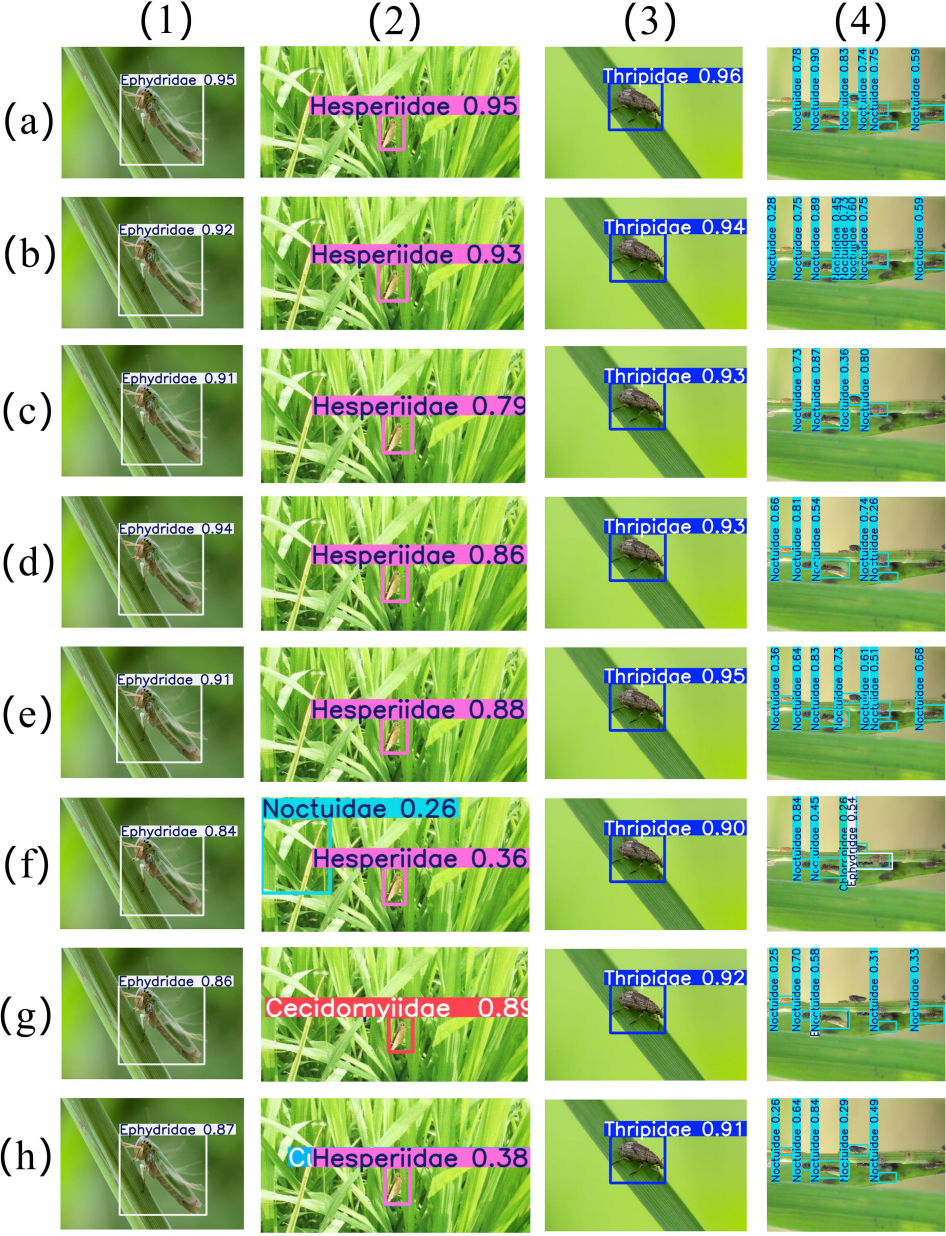
Visualization comparison of different model detection results, where **(A)** represents BEAM-YOLO, **(B)** represents YOLOv11, **(C)** represents YOLOv10, **(D)** represents YOLOv9, **(E)** represents YOLOv8, **(F)** represents YOLOv6, **(G)** represents YOLOv5, **(H)** represents YOLOv3-tiny.

To comprehensively evaluate the practical performance of the BEAM-YOLO model in rice pest detection tasks, this study comparatively analyzed the detection results of this model and mainstream YOLO series models in multiple typical scenarios, as shown in **Figure 15**. The experimental design included four representative detection scenarios: (1) Ephydridae pests in green leaf backgrounds, for evaluating model precision in target recognition under similar backgrounds; (2) tiny Hesperiidae pests on green leaves, for testing the model’s ability to capture fine morphological features; (3) extremely small-sized Thripidae pests, for examining the sensitivity of detection models to minuscule targets; and (4) multiple Noctuidae pests in complex backgrounds, for verifying model robustness in multi-target complex environments. Through these challenging representative scenarios, the algorithm evaluated the performance differences between the BEAM-YOLO constructed based on four innovative modules and existing YOLO variants.

The experimental results intuitively demonstrate the detection advantages of the BEAM-YOLO model in various complex scenarios. In scenario (1), BEAM-YOLO’s detection confidence for Ephydridae pests reached 0.95, higher than other models, particularly 0.21 higher than YOLOv3-tiny; in scenario (2) with extremely small Hesperiidae targets, BEAM-YOLO’s recognition confidence reached 0.95, while YOLOv6 incorrectly identified it as Cecidomyiidae, and YOLOv5 exhibited confusion with double tagging; in scenario (3), all models performed relatively similarly, but BEAM-YOLO still led with the highest confidence of 0.96; in the most challenging scenario (4), BEAM-YOLO successfully detected all Noctuidae targets with more balanced confidence distribution, while YOLOv6 and YOLOv3-tiny misclassified some targets as Ephydridae, and YOLOv10, YOLOv9, and YOLOv5 showed obvious missed detections. These results fully validate the synergistic effect of the innovative modules in BEAM-YOLO: the MEN edge enhancement module precisely captures pest morphological features, the BAFE contrastive wavelet attention effectively separates pest targets from complex backgrounds, and the EM-BFPN feature pyramid network and SCAU upsampling module significantly enhance multi-scale feature extraction and fusion capabilities, giving BEAM-YOLO comprehensive performance advantages in detection tasks involving small targets, similar morphologies, and complex backgrounds like rice pests.

## 5 Conclusion

This study presents BEAM-YOLO, a detection framework that addresses technical bottlenecks in intelligent rice pest detection while achieving an optimal balance between detection accuracy and computational efficiency. The four innovative modules work synergistically to effectively resolve practical application challenges faced by existing algorithms in actual field environments. The MEN module enhances morphological feature capture through multi-scale feature representation and edge enhancement mechanisms. The BAFE module innovatively employs wavelet decomposition combined with a dual attention mechanism, overcoming foreground-background confusion problems in complex agricultural environments. The EM-BFPN network strengthens information exchange between different feature levels through adaptive feature fusion and multi-scale convolution processing. The SCAU upsampling module improves detection sensitivity for small-sized targets by introducing channel shuffling and spatial displacement strategies.

Extensive comparative experiments demonstrate that our proposed model outperforms current state-of-the-art detection algorithms across various evaluation scenarios, showing particularly significant advantages when handling challenging situations such as complex agricultural environments, minute targets, and morphologically similar pests. Future research will focus on exploring model lightweight strategies to enable edge device deployment, integrating temporal information to develop real-time monitoring systems based on video sequences. Additionally, establishing shared data platforms utilizing blockchain technology and promoting industry standards will further facilitate large-scale application of precision agricultural pest control technologies, contributing to food security and sustainable agricultural development.

This study has several limitations that should be acknowledged. First, the JRICE-PD dataset was collected from a single region (Jiangxi Province), which may limit generalization to other geographical areas with different pest species distributions. Second, the dataset exhibits class imbalance (5.4:1 ratio), potentially affecting detection performance on minority classes. Third, all experiments were conducted on static images; real-time performance on video streams and temporal consistency remain to be validated. Fourth, inference latency was not measured on edge devices; preliminary tests on RTX 4060 show 8.2ms per frame, but embedded platform performance requires further investigation.

## Data Availability

The original contributions presented in the study are included in the article/supplementary material. Further inquiries can be directed to the corresponding author.
